# IFI27 enhances bladder cancer immunotherapy response by modulating regulatory T cell enrichment

**DOI:** 10.7150/jca.99014

**Published:** 2024-10-28

**Authors:** Peng Wang, Ning Jiang, Jianye Zhong, Qiwei Chen, Renliang Huang, Chunxiao Liu, Peng Xu

**Affiliations:** 1 Department of Urology, Zhujiang Hospital, Southern Medical University, Guangzhou, China.; 2 Department of Urology, South China Hospital, Medical School, Shenzhen University, Shenzhen, China.

**Keywords:** Bladder cancer, IFI27, Regulatory T cell, FOXP3, Immunotherapy.

## Abstract

Bladder cancer (BCa) is the 10th most prevalent cancer globally. Neoadjuvant therapy has become the standard treatment for muscle-invasive bladder cancer, yet the pathologic complete response rate for patients is only approximately 35%. However, the mechanisms underlying neoadjuvant therapy resistance in bladder cancer patients remain unclear. We collected two sets of paired bladder cancer specimens before and after neoadjuvant therapy, and performed RNA sequencing. The findings revealed a significant decrease in IFI27 expression levels in the post-neoadjuvant therapy group compared to samples collected before treatment, suggesting that IFI27 may play a role in resistance to neoadjuvant combination therapy. IFI27, a member of the interferon-alpha (IFN-α) inducible gene family, influences the efficacy of immune checkpoint blockade therapy. Further analysis demonstrated that IFI27 is predominantly expressed in the cytoplasm of bladder cancer cells and exhibited low expression levels in bladder cancer tissues and cell lines. Subsequently, we investigated the inhibitory effects of IFI27 on bladder cancer proliferation, migration, epithelial-mesenchymal transition, and lymph node metastasis. Additionally, in a mouse model, PD-1Ab immunotherapy was found to upregulate IFI27 while downregulating the protein level of FOXP3, a key transcription factor for regulatory T cells. Flow cytometric analysis further demonstrated that IFI27 inhibits bladder cancer progression by suppressing regulatory T cell infiltration and enhancing anti-tumor immune responses. In conclusion, these findings establish IFI27 as a promising molecular marker for improving the efficacy of immunotherapy in bladder cancer and offer valuable insights into strategies for enhancing immunotherapy sensitivity.

## Introduction

Bladder cancer (BCa) is the 10th most prevalent cancer globally, with a male-to-female incidence ratio of 4:1 and notably higher rates in developed countries 1, 2. Radical cystectomy is the primary surgical treatment for BCa, however, approximately 50% of patients undergoing this procedure experience recurrence and metastasis, resulting in a 5-year post-surgery survival rate of only 15% 3-5. The advent of PD-1/PD-L1 inhibitors has ushered in a new era of immunotherapy for urothelial carcinoma, with several inhibitors now used as first- and second-line treatments for metastatic urothelial carcinoma patients 6. Immunotherapy, as a novel adjuvant treatment, is gradually becoming established in clinical practice. Multiple studies have indicated that preoperative neoadjuvant chemotherapy or immunotherapy can improve survival and tumor downstaging rates in patients with muscle-invasive bladder cancer 7-9. Current clinical response rates for platinum-based neoadjuvant chemotherapy and neoadjuvant immunotherapy alone are approximately 40%-60% and 15%-25%, respectively 10. In cases where neoadjuvant immunotherapy is combined with chemotherapy (e.g., nivolumab with gemcitabine and cisplatin), the pathological complete response (pCR) rate reaches 35% 11. However, a subset of patients remains resistant to these therapies. It is urgent to explore new molecular targets to enhance the efficacy of immunotherapy, which is critical for developing more effective BCa immunotherapies and combination therapies.

IFI27 (interferon alpha inducible protein 27) belongs to the interferon-alpha (IFN-α) inducible small gene family and primarily exerts its biological functions by inducing interferon-stimulated genes (ISGs) 12. IFI27, identified as a proliferation marker in tumors and epithelial cells, plays a role in the progression of various cancers, including cutaneous squamous cell cancer, cholangiocarcinoma, and oral squamous cell carcinoma, and is associated with long-term prognosis 13-15. Epithelial-Mesenchymal Transition (EMT) is a critical process wherein epithelial cells transform into mesenchymal cells, leading to increased mobility and migratory potential. This process plays a crucial role in tumor metastasis and dissemination 16, 17. Research indicates that IFI27 mediates EMT and is involved in the progression of head and neck squamous cell carcinoma as well as nasopharyngeal carcinoma 18, 19.

Regulatory T (Treg) cells are crucial mediators of immune suppression in tumors and accumulate in tumor tissues 20. The transcription factor Forkhead box protein 3 (FOXP3) is a key regulatory factor for the development and function of CD4^+^ CD25^+^ regulatory T cells and plays a crucial role in mediating the immune suppressive activity of Treg cells 21-24. Abundant evidence suggests that an increased presence of endogenous FOXP3^+^ CD25^+^ CD4^+^ Treg cells in tumor tissues is associated with poor prognosis. Additionally, reducing the recruitment of Treg cells in tumors can enhance the anti-tumor immune response 25, 26. Extensive research on various types of tumors, including bladder cancer, has highlighted an increase in the number of Treg cells within the tumor microenvironment (TME) of cancer patients. These findings provide compelling evidence that supports the development of an immune-suppressive TME, which contributes to immune evasion in tumors 27-32. Interferon signaling is a crucial component of immune responses and is essential for modulating the effectiveness of immune checkpoint blockade (ICB) therapy 33.

Recent studies have highlighted IFI27 as a crucial immune regulatory gene, actively participating in the immune response and showing a significant correlation with the frequency of enrichment of CD4^+^ T cells 34, 35. The expression of IFI27 not only affects the response to immunotherapy but also influences various phenotypes related to inflammation and immunity 36, 37. Interestingly, in metastatic breast cancer, there is a coordinated change in the expression of the interferon-regulated gene IFI27 and the abundance of Treg cells. This research offers valuable insights into predictive features of the immune therapy response 38. Nevertheless, the specific influence of IFI27 on Treg cells in bladder cancer (BCa) remains elusive. Given the increasingly prominent regulatory role of IFI27 in Treg cell enrichment within the tumor immune microenvironment, we aimed to explore whether IFI27 exerts a suppressive effect on Treg cell frequency in bladder tumors. This could potentially enhance the anti-tumor immune response. Since IFI27 contributes to the anti-tumor immune response, this study focuses on how IFI27 modulates the immune microenvironment and the development of malignancy in BCa. We aim to explore the specific biological roles of IFI27 and assess its potential as a therapeutic target for BCa.

## Materials and methods

Detailed materials and methods are described in Supplementary file S1.

## Results

### IFI27 is poorly expressed in BCa tissues and cells

**Figure 1:**
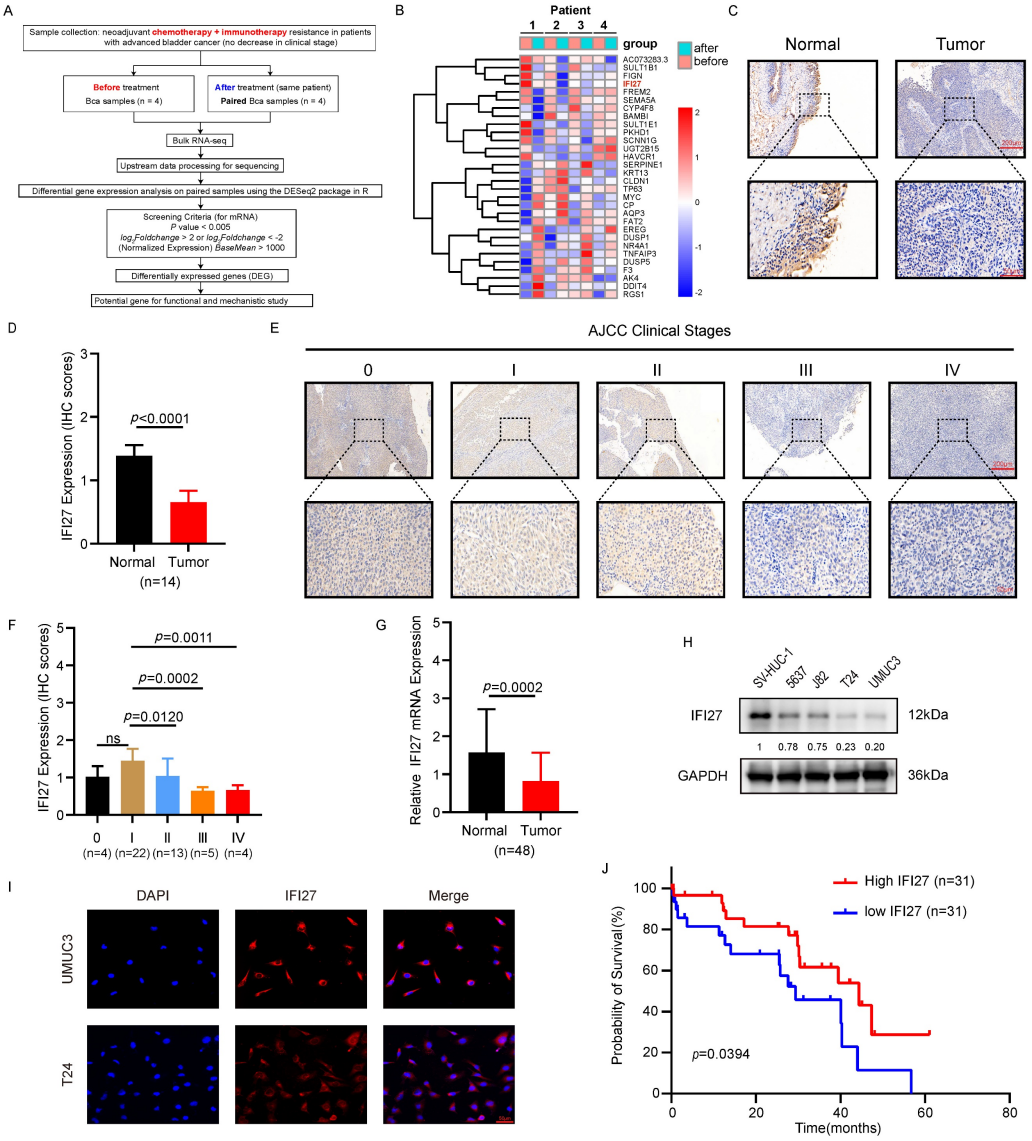
** IFI27 is lowly expressed in BCa tissues and cells. A** Brief process for RNA sequencing of clinical samples.** B** Heatmap showing mRNA levels of different genes expressed in bladder tumor samples before and after chemotherapy combined with immunotherapy as determined by RNA sequencing. **C** Detection of IFI27 protein levels in BCa and paired adjacent tissues through IHC staining. Scale bar =200μm, 50μm. **D** The IHC score of IFI27 expression in **(C)** was quantified.** E**, **F** Description of representative IHC staining images of IFI27 protein in BCa patients with different AJCC clinical stages and statistical analysis of the images. Scale bar = 200μm, 50μm. **G** IFI27 expression level displays significant variation in BCa tissues versus adjacent normal tissues (n=48). **H** Western blotting was conducted to estimate the protein levels of IFI27 in control SV-HUC-1 and BCa cell lines 5637, J82, T24, and UM-UC-3 cells. **I** IFI27 was mainly localized in the cytoplasm of BCa cells by immunofluorescence analysis. Scale bar =50μm. **J** Low IFI27 expression level implies poor prognosis in BCa patients from our institution.

To elucidate the molecular mechanisms underlying resistance to chemotherapy combined with immunotherapy in BCa patients, we procured paired samples from four late-stage bladder cancer patients both before and after administration of the combined treatment. RNA sequencing was subsequently performed to identify differentially expressed genes. The heatmap results indicated that the expression pattern of the top-ranked gene, AC073283.3, was inconsistent in the third pair of matched samples compared to the other pairs. Similarly, the third-ranked gene, FIGN, exhibited an inconsistent expression pattern in the fourth pair of samples. The second-ranked gene, SULT1B1, a histone sulfotransferase, was primarily involved in biometabolic catabolic processes and has no reported role in tumor immunotherapy 39, 40. In contrast, IFI27, a gene associated with the interferon pathway, was closely linked with tumor development and the regulation of T lymphocytes in the immune microenvironment 41. RNA sequencing results showed a significant decrease in IFI27 expression levels in the post-neoadjuvant treatment group compared to pre-treatment samples, suggesting that low IFI27 expression may correlate with resistance to chemotherapy combined with immunotherapy in BCa patients. Therefore, based on these findings, IFI27 was selected as the focus of our subsequent research (Fig. 1 A-B). Given its crucial role in modulating the immune response and influencing the effectiveness of tumor immunotherapy 34, 36, we further investigated the correlation between IFI27 and clinical features of BCa. Immunohistochemistry (IHC) analysis confirmed reduced IFI27 expression in BCa tissue compared to adjacent normal bladder tissue (Fig. 1 C-D). Bladder tumor samples were classified into stages 0, I, II, III, and IV according to the AJCC clinical staging system. IFI27 expression was downregulated in late-stage BCa (stages III and IV) compared to early stages (stages 0, I, and II) (Fig. 1 E-F). Analysis of IFI27 mRNA in clinical samples from Zhujiang Hospital also indicated reduced expression compared to adjacent normal bladder tissue (Fig. 1 G). We also explored the correlation between IFI27 and clinical characteristics of BCa patients. The results showed that IFI27 was significantly correlated with tumor clinical stage, tumor infiltration, lymph node metastasis, and distant metastasis (**Table 1**). Furthermore, IFI27 protein levels were markedly lower in BCa cell lines (5637, J82, T24, and UM-UC-3) compared to the normal human bladder epithelial cell line SV-HUC-1 (Fig. 1 H). Immunofluorescence staining revealed that IFI27 was predominantly localized in the cytoplasm of BCa cells (Fig. 1 I). Kaplan-Meier analysis of 62 BCa cases indicated that high IFI27 expression was associated with improved survival rates (Fig. 1 J). Overall, IFI27 exhibited low expression levels in BCa tissues and cells, and this low expression may be related to resistance to post-neoadjuvant therapy in BCa patients.

### IFI27 suppresses the proliferation and migration capability of BCa cells

**Figure 2:**
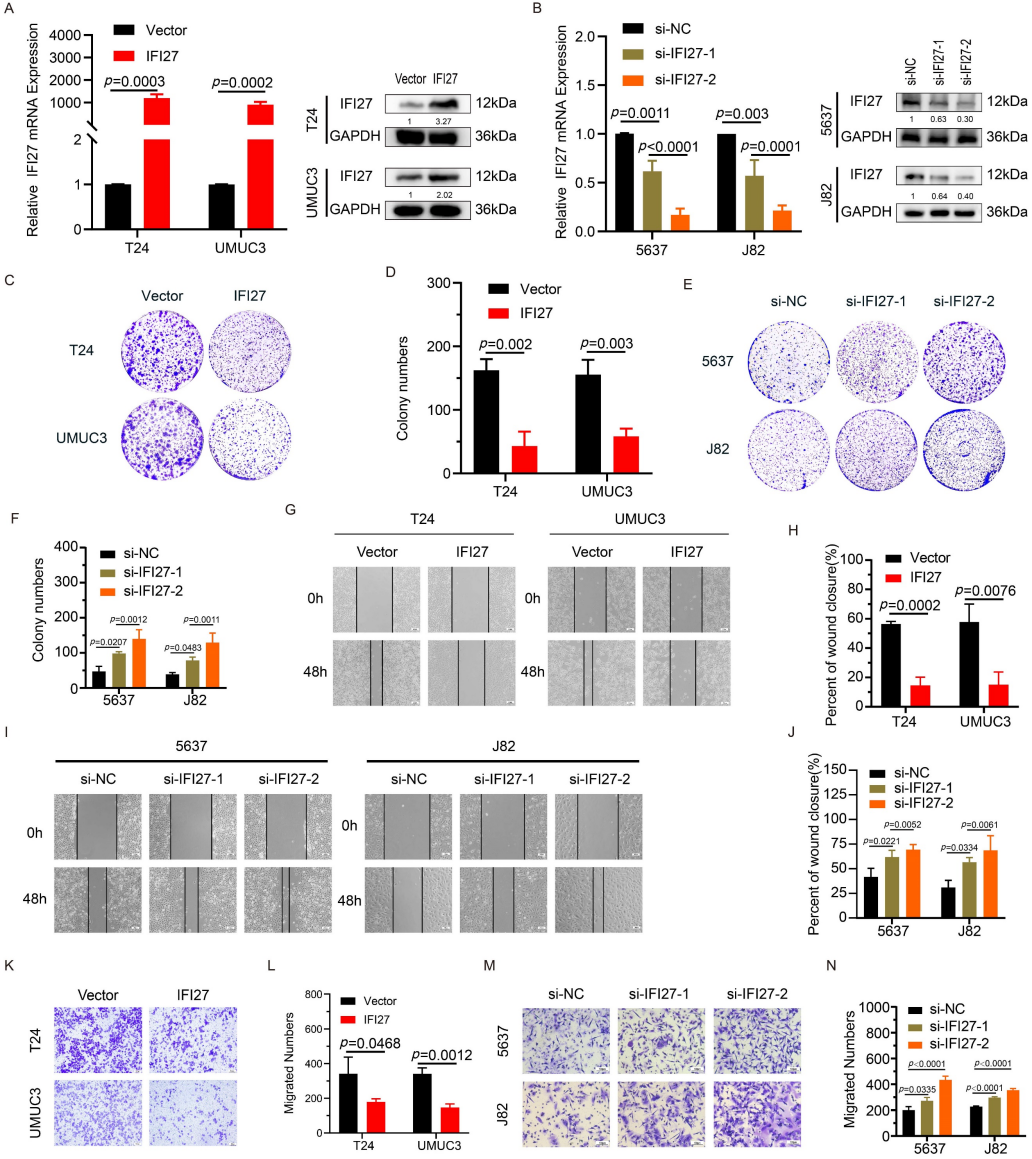
** IFI27 inhibits BCa cell proliferation, migration. A** qRT-PCR and western blotting showed the efficiency of IFI27 overexpression in T24 and UM-UC-3 cells infected by lentivirus. **B** Knockdown efficiency of IFI27 after transient transfection of 5637 and J82 cells as determined by qRT-PCR and western blotting. **C**, **D** Colony formation in T24 and UM-UC-3 cells with IFI27 overexpression along with the corresponding statistical analysis results. **E**, **F** The colony-forming ability of 5637 and J82 cells after IFI27 knockdown was changed and shown as a statistical graph. **G**, **H** Wound-healing experiments indicated changes in the migratory ability of T24 and UM-UC-3 cells with IFI27 overexpression, and the representative images were quantified by statistical analysis. **I**, **J** Changes in the migratory capacity of 5637 and J82 cells after IFI27 knockdown were demonstrated by wound-healing assay and measured by statistical analysis. **K**, **L** Transwell assay revealed altered migratory ability of T24 and UM-UC-3 cells overexpressing IFI27, and representative images were statistically figured. **M**, **N** Transwell assays were performed to verify the altered migratory ability of 5637 and J82 cells knocking down IFI27, and the results were present in statistical plots.

To validate the impact of IFI27 on BCa cell phenotypes, we performed gene knockdown and overexpression experiments in BCa cell lines (5637, J82, T24, and UM-UC-3). T24 and UM-UC-3 cells were transduced with viral vectors to overexpress IFI27, while 5637 and J82 cells underwent IFI27 knockdown using siRNAs. The validation results of IFI27 knockdown or overexpression efficiency were presented (Fig. 2 A-B). Next, we evaluated the effect of IFI27 on the biological functions of BCa cells. Clone formation experiments showed that overexpression of IFI27 inhibited the proliferation of T24 and UM-UC-3 cells, while IFI27 knockdown in 5637 and J82 cells enhanced their proliferation (Fig. 2 C-F). Additionally, wound-healing assays were used to assess the impact of IFI27 on BCa cell migration. Overexpression of IFI27 impaired migration, while IFI27 knockdown significantly enhanced it (Fig. 2 G-J). Similarly, transwell assays produced comparable results for cell migration (Fig. 2 K-N). In summary, IFI27 acted as an inhibitor of both proliferation and migration in BCa cells.

### IFI27 regulates epithelial-mesenchymal transition in BCa cells

**Figure 3:**
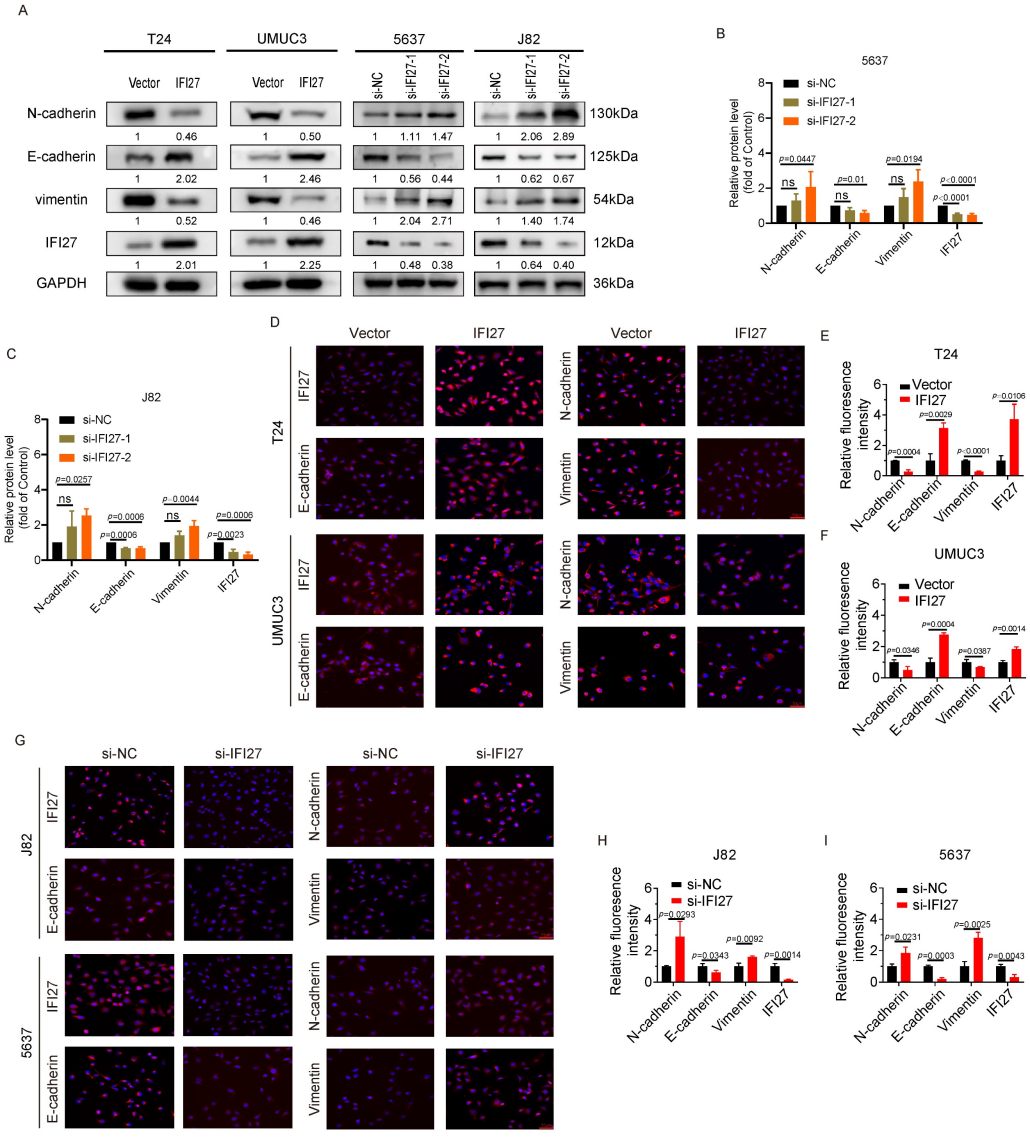
** IFI27 expression level correlates with EMT in BCa cells. A** Four types of Bca cells (T24, UM-UC-3, 5637, J82) were treated with IFI27 knockdown or overexpression. The mesenchymal markers N-cadherin and vimentin and the epithelial marker E-cadherin were detected by western blotting. GAPDH was utilized as the internal control.** B**,** C** Transfection of si-IFI27-2 significantly more effectively affected EMT-associated protein levels in 5637 and J82 cells compared to si-IFI27-1 transfection. **D** Immunofluorescence assays was applied to determine the expression levels of E-cadherin, N-cadherin, Vimentin, and IFI27 in T24 and UM-UC-3 cells with IFI27 overexpression. Scale bar =50μm. **E**,** F** Statistical plots of representative images from immunofluorescence experiments conducted on T24 and UM-UC-3 cells. **G** The expression levels of E-cadherin, N-cadherin, Vimentin and IFI27 were measured by immunofluorescence assay in J82 and 5637 cells transfected si-IFI27-2. Scale bar = 50μm. **H**,** I** Statistical quantification of E-cadherin, N-cadherin, Vimentin and IFI27 expression levels in J82 and 5637 cells.

Epithelial-Mesenchymal Transition (EMT) is a crucial cellular program involved in processes such as embryonic development and malignant tumor progression. In the field of tumor research, EMT can promote the tumorigenicity and metastatic potential of cancer cells, making it a key driving factor in tumor progression 42, 43. Immunostaining experiments provided key insights into the effects of IFI27 on EMT markers. Overexpression of IFI27 in T24 and UM-UC-3 cells resulted in upregulation of the epithelial marker E-cadherin and downregulation of the mesenchymal markers N-cadherin and Vimentin. Conversely, IFI27 knockdown in 5637 and J82 cells led to a decrease in E-cadherin expression and an increase in N-cadherin and Vimentin expression (Fig. 3 A). SiRNAs were used for IFI27 knockdown, with si-IFI27-2 demonstrating the highest efficiency (Fig. 3 B-C). Immunofluorescence assays confirmed these findings, showing increased E-cadherin and decreased N-cadherin and Vimentin expression in T24 and UM-UC-3 cells with IFI27 overexpression (Fig. 3 D-F). Conversely, in 5637 and J82 cells with IFI27 knockdown using si-IFI27-2, the expression patterns of EMT-related proteins were reversed (Fig. 3 G-I). These fluorescence staining observations were consistent with the results obtained from western blotting. Taken together, these findings demonstrated that IFI27 impedes the epithelial-mesenchymal transition process in BCa cells by modulating the expression of EMT-related markers.

### Overexpression of IFI27 inhibits tumor progression and lymph nodes metastasis of BCa *in vivo*

**Figure 4:**
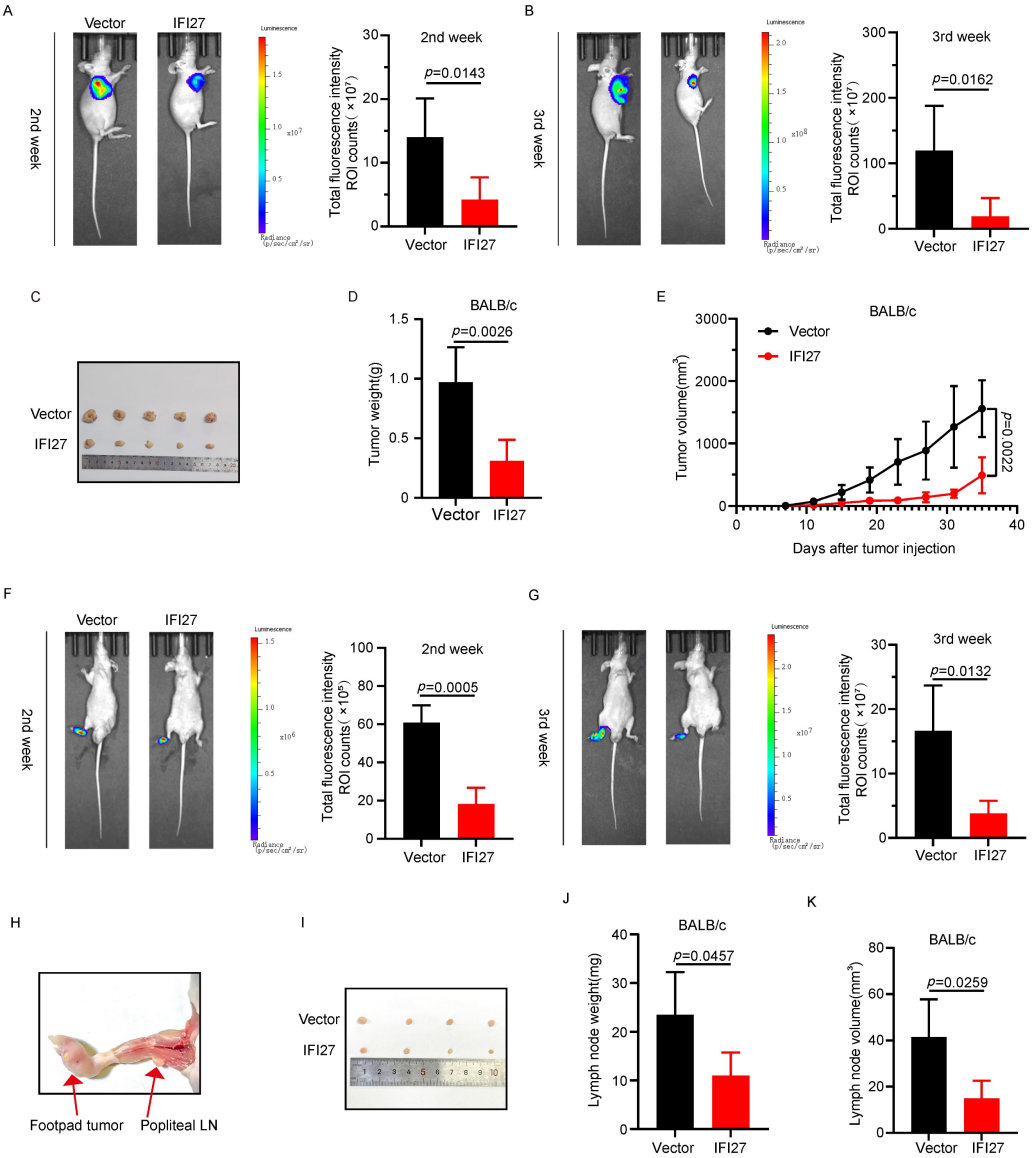
** IFI27 overexpression attenuates tumorigenicity and lymph node metastasis *in vivo*. A**,** B** The progression of the subcutaneous tumor was tracked by an IVIS, and typical photographs of subcutaneous tumors were obtained. Total fluorescence intensity region of interest ROI counts was proceeded to detect the cellular activity of subcutaneous tumors at 2nd **(A)** and 3rd **(B)** week post-injection (n = 5 per group).** C** Representative images showcased subcutaneous tumor harvested after execution of nude mice. **D**,** E** Statistical analysis of tumor weight **(D)** and volume **(E)** in mice.** F**,** G** Total fluorescence intensity ROI counts were used to detect tumor growth and lymph node metastasis at 2nd **(F)** and 3rd **(G)** week after injection (n = 4 per group). **H**,** I** Schematic diagram **(H)** and representative images **(I)** of popliteal lymph nodes in mice. **J**,** K** Statistical analysis of popliteal lymph node weight **(J)** and volume **(K)** in mice.

To confirm the impact of IFI27 on tumorigenesis *in vivo*, we injected T24 cells overexpressing IFI27 or control T24 cells into the axillary region of nude mice to establish a subcutaneous xenograft model. Representative bioluminescence images taken two to three weeks after injection showed that IFI27 overexpression inhibited tumor growth compared to the control group (Fig. 4 A-B). At day 28, mice were euthanized, and subcutaneous tumors were harvested, with representative images of the tumor tissues presented (Fig. 4 C). Statistical analysis of tumor weight and volume revealed that IFI27 overexpression suppressed xenograft tumor growth, resulting in reduced tumor weight and volume compared to controls (Fig. 4 D-E). Tumor metastasis to lymph nodes (LN) often signifies a poor prognosis for individuals with advanced stages of BCa 44, 45. To assess the potential role of IFI27 in BCa lymph node metastasis *in vivo*, we inoculated tumor cells into the footpad of nude mice, establishing a footpad-popliteal lymph node model. Two weeks after tumor cell injection, bioluminescence imaging showed a lower footpad signal in the IFI27 overexpression group compared to the control group, although signals in the popliteal lymph nodes were barely detected. By the third week, the IFI27 overexpression group exhibited lower bioluminescence intensity in the popliteal lymph nodes compared to the control group (Fig. 4 F-G). At four weeks, mice were euthanized, and popliteal lymph nodes were dissected for analysis (Fig. 4 H-I). Volume and weight measurements of these lymph nodes indicated that IFI27 overexpression attenuated tumor lymphatic metastasis (Fig. 4 J-K). In short, upregulation of IFI27 expression suppressed bladder tumor proliferation and lymph node metastasis *in vivo*.

### IFI27 overexpression reinforces anti-tumor immunity

**Figure 5:**
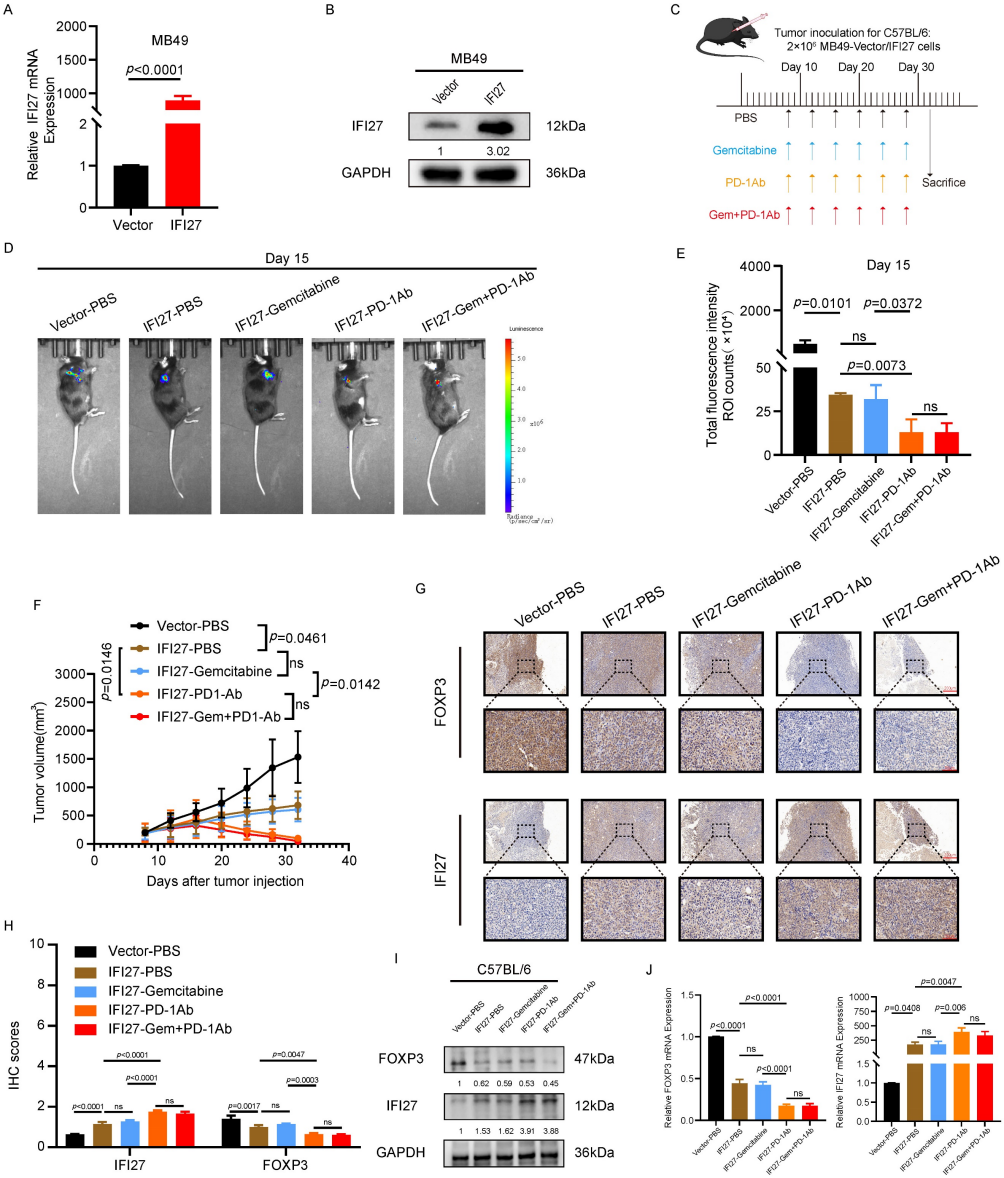
** Overexpression of IFI27 enhances the anti-tumor immune response. A**,** B** The overexpression efficiency of IFI27 in MB49 cells was verified by qRT-PCR **(A)** and western blotting **(B)**. **C** Schematic diagram of the therapeutic process of drug administration in mice. **D, E** Representative diagrams of subcutaneous tumor biofluorescence in mice **(D)**, and statistical analysis of biofluorescence intensity in each group of mice on 15th day after injection **(E)**. **F** Statistical analysis of volume in mice tumor. **G**,** H** The expression levels of FOXP3 and IFI27 in tumor tissues of different experimental animal groups were detected by IHC **(G)**, and the captured representative images were subjected to immunohistochemical scoring for statistical analysis **(H)**. Scale bar = 200μm, 50μm.** I**,** J** The expression levels of IFI27 and FOXP3 in mouse tumor tissues were examined by western blotting **(I)** and qRT-PCR **(J)**.

To investigate the effect of IFI27 on the efficacy of chemotherapy combined with immunotherapy for BCa, we established an animal subcutaneous tumor model using the murine BCa cell line MB49. IFI27 overexpression in MB49 cells was achieved using viral vectors, and the efficiency of overexpression was verified at the protein and mRNA levels (Fig. 5 A-B). Immunocompetent mice were randomly assigned to different groups and then injected with MB49 tumor cells to establish the subcutaneous tumor model. On day 8 after tumor implantation, drug treatments were administered via intraperitoneal injection every four days for a total of six doses (Fig. 5 C). The *in vivo* imaging system (IVIS) was utilized to detect subcutaneous tumor formation. Statistical analysis of bioluminescence intensity revealed significant inhibition of tumor proliferation in immunocompetent mice with IFI27 overexpression. In the MB49 tumor model with IFI27 overexpression, gemcitabine treatment alone did not show a statistically significant difference in tumor growth compared to the PBS control group. PD-1 antibody immunotherapy significantly inhibited tumor progression compared to gemcitabine monotherapy. However, no significant difference in efficacy was observed between the combination of PD-1 antibody and gemcitabine and PD-1 antibody alone (Fig. 5 D-E). Tumor volume measurements supported these findings with statistical significance (Fig. 5 F). Immunoinfiltration analysis of TCGA database data showed no significant difference in the enrichment and infiltration of activated CD8^+^ T cells between normal bladder tissues and BCa tissues. However, there was a statistically significant difference in the enrichment and infiltration of activated CD4^+^ T cells between these tissues (Fig. S1A-B). A study published in the journal *Cell* reported that the typical CD8^+^ T cell state was not enriched in the bladder tumor microenvironment and the frequency of CD4^+^ T cells was higher than that of CD8^+^ T cells in BCa 46. These findings prompted us to shift our research focus to the CD4^+^ T-cell subset within the bladder tumor microenvironment. Regulatory T cells (Tregs) with elevated abundance negatively impact the anti-tumor immune response in BCa 47. These Tregs, characterized by persistent expression of FOXP3, CD25, and CD4, exhibit immunosuppressive capabilities, inducing immune tolerance and fostering immunosuppression within the T-cell population 48, 49. Additionally, Tregs were enriched in both BCa tissues and peripheral blood 50. Considering the above findings, our next research goal was to investigate whether IFI27 was involved in the anti-tumor immune response by regulating Tregs infiltration in the BCa microenvironment. To investigate whether FOXP3-expressing Treg cells were involved in the promotion of anti-tumor immunity by IFI27, immunohistochemical staining was conducted on collected tumor tissues. The results showed that PD-1 antibody immunotherapy increased IFI27 protein expression and decreased FOXP3 expression compared to the IFI27-PBS control. In contrast, gemcitabine monotherapy did not significantly alter IFI27 or FOXP3 protein expression levels compared to the combination of gemcitabine and PD-1 antibody immunotherapy (Fig. 5 G-H). Proteins and mRNA derived from mouse tumor tissues validated the above findings (Fig. 5 I-J). These results collectively demonstrated that IFI27 overexpression enhanced the efficacy of BCa immunotherapy. However, in the IFI27-overexpressing mouse tumor model, the efficacy of PD-1 antibody monotherapy did not differ significantly from that of the combination therapy with gemcitabine and PD-1 antibody.

### IFI27 augmented tumor immunity via inhibition of Treg *in vivo*

**Figure 6:**
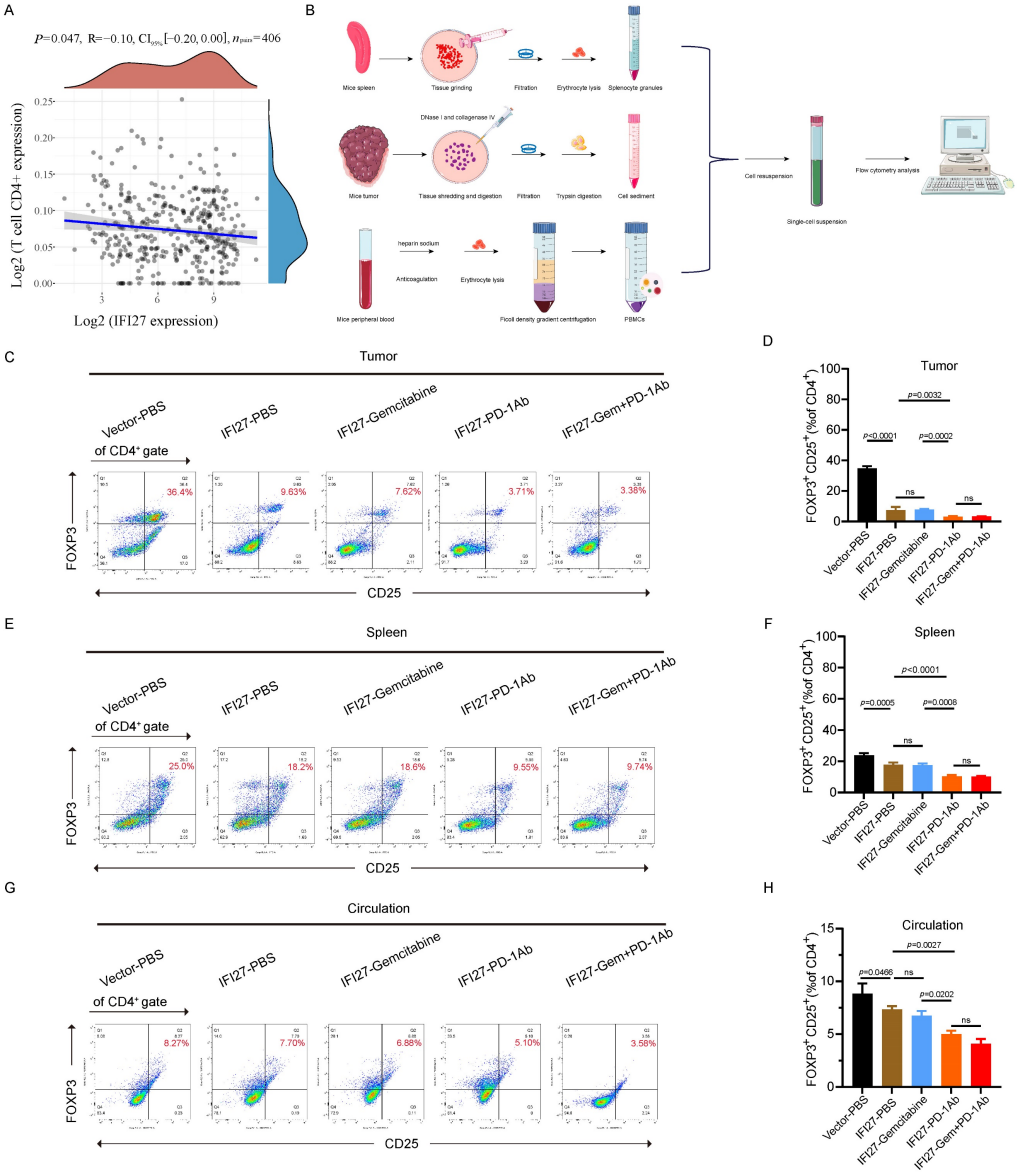
** IFI27 is involved in tumor immunity by inhibiting the enrichment of regulatory T cells. A** FOXP3 showed a negative correlation with CD4^+^ T cell enrichment in BCa. **B** Schematic diagram of the preparation of mouse tumor tissue, spleen, and peripheral blood single-cell suspension. **C**-**H** Flow cytometry representative graphs demonstrated the proportion of Treg cells in mouse tumor** (C)**, spleen **(E)** and peripheral blood** (G)**. Statistical analysis showed the differences in Treg cells enrichment in tumor **(D)**, spleen **(F)** and peripheral blood **(H)** of mice in each group.

Indeed, in BCa, IFI27 significantly correlated with a variety of immune cell subsets (Fig. S2). Although IFI27 correlated well with CD8^+^ T cell populations, these populations were not significantly enriched in BCa, as previously described. This led us to focus more on the infiltration of CD4^+^ T cell subpopulations, particularly Treg cell subpopulations, into the BCa immune microenvironment. Clinical data from BCa patients in the TCGA database confirm that FOXP3 was not only significantly negatively correlated with the enrichment of CD4^+^ T cells (Fig. 6A), but also significantly associated with Treg cells (Fig. S2). To further validate the involvement of Treg cells in the IFI27-mediated promotion of anti-tumor immunity, we collected mouse spleens, tumor tissues, and peripheral blood. Single-cell suspensions were prepared following the experimental protocol for flow cytometry antibody staining (Fig. 6 B). In mice tumor tissues, there was no significant difference in CD8^+^ T cell infiltration following PD-1 antibody immunotherapy compared to the IFI27-PBS control group (Fig. S3A-B). However, CD4^+^ T cell enrichment was reduced (Fig. S3 A, C), and Treg cell infiltration was also minimized (Fig. 6 C-D). Similarly, compared to gemcitabine chemotherapy, PD-1 antibody immunotherapy demonstrated no significant difference in CD8^+^ T cell infiltration in tumor tissue (Fig. S3A-B), but CD4^+^ T cell enrichment diminished (Fig. S3 A, C) and Treg cell frequency also decreased (Fig. 6 C-D). Furthermore, the combination of gemcitabine chemotherapy and PD-1 antibody immunotherapy did not significantly alter the frequencies of CD8^+^ T cells, CD4^+^ T cells, and Treg cells versus PD-1 antibody immunotherapy alone (Fig. S3 A-C and Fig. 6 C-D). Similar phenomena were observed in spleen tissues (Fig. S3 D-F and Fig. 6 E-F) and peripheral blood (Fig. 6 G-H). In brief, IFI27 boosted anti-tumor immune responses in BCa. This was achieved by down-regulating Treg cells enrichment in the tumor microenvironment.

### Correlation of IFI27/PD-1/FOXP3 expression with the clinicopathological characteristics of BCa

**Figure 7:**
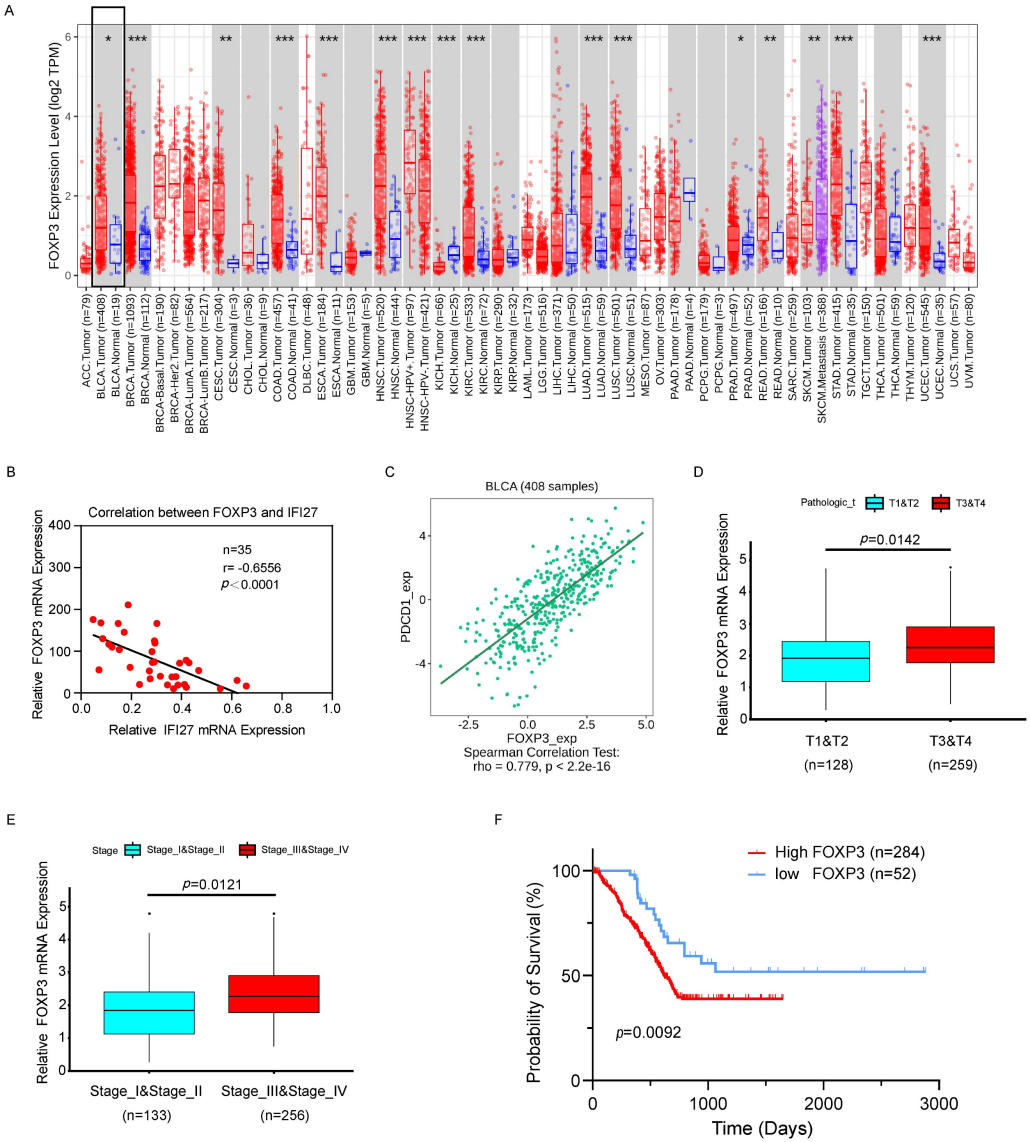
** FOXP3 validation and expression in BCa. A** Pan-cancer differential analysis revealed that FOXP3 was highly expressed in BCa tissues compared to normal bladder tissues. **B** Negative correlation between IFI27 and FOXP3 expression was demonstrated in BCa tissues collected from our institution (n=35, r=-0.6556, *p*<0.0001). **C** PDCD1 (PD-1) presented a positive correlation with FOXP3 expression in BCa. **D**-**E** FOXP3 expression showed a positive correlation with the **(D)** tumor infiltration stages (n_T1+T2_ = 128, n_T3+T4_ = 259**)** and **(E)** clinical stages (n_stage I+II_ = 133, n_stage III+IV_ = 256**)** in BCa patients. **F** High FOXP3 expression predicts poor prognosis in BCa patients (n_high_ = 284, n_low_ = 52).

The clinical correlation of PDCD1 (PD-1) and FOXP3 was analyzed using samples from our institution and data from TCGA. Initially, a representative graph illustrating high FOXP3 expression in BCa tissues was obtained from TIMER 2.0 (http://timer.comp-genomics.org/timer/) (Fig. 7 A). Furthermore, correlation analysis of IFI27 and FOXP3 expression in BCa tissue samples from 35 patients revealed a negative correlation, with a Pearson r of -0.6556 (*p* < 0.0001; Fig. 7 B). These findings further validated the negative correlation between IFI27 and FOXP3 expression in BCa tissues. Subsequently, TCGA database samples indicated a positive correlation between PD-1 and FOXP3 expression (http://cis.hku.hk/TISIDB/index.php) (Fig. 7 C). Further analysis of the relationship between FOXP3 and tumor infiltration stages revealed that FOXP3 abundance was up-regulated at T3 and T4 stages compared to T1 and T2 stages in BCa (Fig. 7 D). Subsequent analysis of the relationship between FOXP3 and clinical stages in BCa showed an significant increase in FOXP3 abundance in advanced stages (Fig. 7 E). In a survival analysis of 336 cases of TCGA-database patients, high FOXP3 expression predicted a poor prognosis in advanced stages (*p*=0.0092; Fig. 7 F). Besides, we employed BCa cell lines to investigate the expression levels of FOXP3 following overexpression or knockout of IFI27. Western blot analysis indicated that in BCa cell lines T24 and UM-UC-3, where IFI27 was overexpressed, the expression levels of FOXP3 were significantly reduced (Fig. S4 A). Conversely, in BCa cell lines 5637 and J82 with down-regulated IFI27 expression, FOXP3 levels were upregulated (Fig. S4 B). All in all, the correlation between IFI27 expression and FOXP3 was supported by clinical samples and TCGA BCa patient data, suggesting that the IFI27/PD-1/FOXP3 axis was involved in the modulation of anti-tumor immune processes in BCa.

## Discussion

The biological regulatory functions of interferon pathway-related genes in the tumor immune microenvironment are receiving increasing attention 51, 52. IFI27, as an interferon-alpha-induced protein, is closely associated with tumor occurrence and the regulation of T lymphocytes within the immune microenvironment 41, 53. Previous studies have confirmed IFI27 as a critical immune regulatory gene, promoting cancer immunity by increasing the sensitivity of tumor cells to the cytotoxic effects of human immune cells, thus exerting an inhibitory effect on cancer development 54. These findings support our perspective on IFI27 as a bladder cancer suppressor in our research.

As a characteristic gene of regulatory T cells (Tregs), FOXP3 has received increasing attention for its regulatory role in tumorigenesis 55, 56. In non-small cell lung cancer, FOXP3 activated the downstream Wnt pathway through the interaction of β-catenin and TCF4, promoting tumor proliferation and metastasis 57. Furthermore, FOXP3 inhibits oncogenic ErbB2 overexpression by binding to the ErbB2 promoter, which ultimately inhibits breast cancer progression 58. Additionally, FOXP3 is an independent prognostic factor in breast cancer, with high expression levels indicating a poor prognosis in late-stage patients 59. Likewise, the expression of FOXP3 is inversely correlated with long-term survival in bladder cancer 60. The above points highlighted the important regulatory role of FOXP3 in tumorigenesis. In addition, we also verified the regulatory role of IFI27 on FOXP3 in BCa cell models. These points suggest that IFI27 may be involved in regulating FOXP3 expression in bladder cancer, which requires further studies in the future.

The notion that regulatory T cells (Tregs) play a crucial role in tumor infiltration and proliferation is gradually emerging 61-63. Reports on various malignant tumors, including BCa, suggest an increased accumulation of Tregs in both tumor tissue and peripheral blood 47, 50, 64. Inhibiting Treg cell regulators such as FOXP3, and thereby reducing the infiltration of tumor Treg cells, becomes an effective approach to enhance anti-tumor immunity and suppress tumor growth 65, 66. In this study, we found an association between FOXP3 and IFI27 in a mouse model, suggesting potential direct or indirect interactions in specific molecular pathways. While FOXP3 is recognized as a regulatory gene in T cells, IFI27, an interferon-related gene, may play a role in inflammatory responses and immune regulation 41. On one hand, we extracted proteins from mouse tumor tissues, and on the other, we prepared single-cell suspensions from mouse tumor, spleen, and peripheral blood. Through immunoblotting and flow cytometry, we confirmed that IFI27 regulates FOXP3 expression in Treg cells. Put it briefly, FOXP3 in Treg cells was used as a downstream marker of IFI27 to explore the specific regulatory mechanisms involved in BCa. Moreover, targeted interventions aimed at this mechanism could eventually be applied in the clinical treatment of BCa. In summary, we demonstrated that overexpression of IFI27 inhibits BCa growth by reducing the infiltration of Treg cells in the tumor parenchyma through the suppression of FOXP3 expression in Treg cells.

Despite the success of immune checkpoint-targeted immunotherapy in various cancers 67-70, BCa patients still exhibit limited sensitivity to immune checkpoint therapy, chemotherapy, and their combination 10, 11, 71. Based on these observations, our *in vivo* experiments investigated how enhanced IFI27 expression affects the efficacy of BCa immunotherapy and chemotherapy. The results demonstrate that PD-1 antibody therapy effectively hinders the infiltration of Treg cells in the tumor stroma, thereby inhibiting tumor growth. Moreover, IFI27 overexpression enhances the inhibitory effect of PD-1 antibody immunotherapy, with a more pronounced suppression of tumor growth. Interestingly, in the mouse tumor model with IFI27 overexpression, the efficacy of PD-1 antibody monotherapy was not significantly different from that of the combination therapy with gemcitabine and PD-1 antibody. Despite gemcitabine's inability to induce Treg-mediated immune suppression 72, the reasons for the limited enhancement of efficacy when combining IFI27 with PD-1 antibody immunotherapy and gemcitabine remain unclear. Further research into the underlying molecular mechanisms is warranted.

In summary, IFI27, identified as a BCa suppressor, is closely associated with clinical features such as disease stage and patient survival. Moreover, IFI27 can modulate the cellular phenotypes of BCa cells, including proliferation, migration, and EMT. *In vivo* results show that IFI27 inhibits the Treg cell-specific marker FOXP3, thereby hindering Treg cell recruitment to the tumor and immune organs. This ultimately enhances anti-tumor immune responses and reduces tumor growth. Furthermore, we propose that IFI27 enhances the efficacy of PD-1 antibody immunotherapy, underscoring its role in improving BCa immunotherapy outcomes. Hence, our research provides a theoretical basis for targeting IFI27 in combination with PD-1 antibody immunotherapy, offering a potential new strategy for BCa treatment.

## Conclusion

In conclusion, IFI27 exhibits anticancer effects in bladder tumors by inhibiting tumor growth and preventing the epithelial-mesenchymal transition (EMT). Additionally, IFI27 contributes to anti-tumor immune responses by reducing the infiltration of regulatory T cells (Treg) into the tumor stroma. Our findings confirm that targeting IFI27 can effectively enhance the efficacy of immunotherapy, providing new insights into improving the sensitivity of BCa immunotherapy.

## Supplementary Material

Supplementary figures and tables.

## Figures and Tables

**Table 1:** Correlation between IFI27 expression and clinical characteristics in BCa patients.

	No. of patients	IFI27 expression		*p* value
		Low	High	
All patients	62	26	36	
Age (years)				0.6073
<65	30	14	16	
≥65	32	12	20	
Gender				>0.9999
Male	47	20	27	
Female	15	6	9	
Tumor size				0.1095
<2cm	22	6	16	
≥2cm	40	20	20	
Grade				0.1705
Low	10	2	8	
High	52	24	28	
Stage				<0.0001
0~II	50	14	36	
III~IV	12	12	0	
T Infiltrate (T)				<0.0001
Ta~T1	33	6	27	
T2~T4	29	20	9	
Lymphatic metastasis (N)				0.0013**
Yes	7	7	0	
No	55	19	36	
Distant metastasis (M)				0.0268*
Yes	4	4	0	
No	58	22	36	

Chi-square test. *P < 0.05. **P < 0.01. ****P < 0.0001. Staining scores: 0, no staining; 1, weak staining; 2, medium staining; 3, strong staining. Classification criteria: High IFI27 expression: 2,3; Low IFI27 expression: 0,1.

## Abbreviations

BCa: bladder cancer
IFI27: interferon alpha inducible protein 27
EMT: Epithelial-mesenchymal transition
IHC: immunohistochemistry
qRT-PCR: Quantitative real-time polymerase chain reaction
Treg cells: regulatory T cells
PD-1: Programmed cell death protein 1
TME: tumor microenvironment
ICB: immune checkpoint blockade
FOXP3: Forkhead box protein 3
PD-L1: Programmed death-ligand 1
ISGs: interferon-stimulated genes
LN: lymph node
siRNA: Small interfering RNA
TCGA: The Cancer Genome Atlas
