# Supplementary material for: IFI27 enhances bladder cancer immunotherapy response by modulating regulatory T cell enrichment
Source: J Cancer. 2024 Oct 28;15(20):6616–30. doi: 10.7150/jca.99014 (PMC11632990; doi:10.7150/jca.99014)
Supplement: Supplementary file 1 — Supplementary figures and tables. [file jcav15p6616s1.pdf]

MATERIALS AND METHODS

RNA-sequencing (RNA-seq)

Tumor samples were collected from patients diagnosed with advanced bladder cancer. These patients then received a combination of neoadjuvant chemotherapy and immunotherapy, specifically Gemcitabine and PD-1Ab. Following surgical intervention, tumor samples were obtained from four patients who have exhibited resistance to the treatment, as evidenced by failure of tumor downstaging. Next, paired samples collected from four patients before and after receiving chemotherapy combined with immunotherapy were subjected to RNA-seq analysis, aiming to identify genes exhibiting differential expression pre- and post-treatment with the combined regimen. Paired analysis and screening for significantly different genes were conducted using the DEseq2 package in R language, employing the following criteria: DEG$Group[which((nrDeG$pvalue < 0.005)& (nrDEG$log2Foldchange > logrc_cutoff)& (nrDEG$baseMean > 1000))]= "upregulated"; DEG$Group[which((nrDEG$pvalue < 0.005)& (nrDEG$1og2Foldchange < -logFc_cutoff) & (nrDEG$baseMean > 1000))]= "downregulated". The screening results of genes were displayed in the form of a heatmap.

Cell culture

The human BCa cell lines (T24, UM-UC-3, 5637, J82), human normal epithelial cell line, SV-HUC-1 and mouse BCa cell line, MB49, utilized in the experiments were obtained from ATCC (Manassas, USA). Each cell line was cultured individually in the respective basal media, supplemented with 10% fetal bovine serum and 1% penicillin-streptomycin solution. All procedures involving these cell lines were conducted within a biological safety cabinet, and the cells were maintained in a cell

incubator at a constant temperature of 37°C with a carbon dioxide concentration of 5%.

Clinical sample collection

110 cases of BCa samples and 62 cases of adjacent normal tissue samples were obtained from Zhujiang Hospital, Southern Medical University. Prior to surgery, informed consent was obtained from all patients. The collection of samples received approval from the Medical Ethics Committee of Zhujiang Hospital. Tissue specimens were immersed in RNA later Solution (Invitrogen, USA) and stored at -80°C.

Transfection and infection assays

Two short interfering RNAs (siRNAs) were synthesized by Kidan Bioscience (Guangzhou, China) to knock down the target gene IFI27. Lipofectamine 2000 (Invitrogen, USA) was e mployed for their transfection. Lentiviral infection was used to overexpress IFI27 in bladd er cancer cell lines using the vector LV5-CMV-GFP-T2A-puro (GenePharma, China). Cells stably expressing IFI27 were screened by continuous puromycin treatment for 4 days. The siRNA sequences used are as follows: si-IFI27-1: sense: 5'-GGAUUGCUACAGUUGUGAU UG-3', antisense: 5'-AUCACAACUGUAGCAAUCCUG-3'; and si-IFI27-2: sense: 5'-GAGG AUCUCUUAOUCUCUAGG-3', antisense: 5'-UAGAGAGUAAGAGAUCCUCAA-3'.

Quantitative real-time polymerase chain reaction (qRT-PCR)

Total cellular RNA was extracted using RNAiso Plus (TaKaRa, Japan). For mRNA quantificat ion, qRT-PCR assays were conducted on a Stratagene Mx3000P PCR device (Agilent Technol

ogies, California, USA) using Prime Script RT Master Mix (TaKaRa, Japan) and TB Green P remix Ex Taq II (Tli RNaseH Plus) (TaKaRa, Japan). The primer sequences were synthesized by Ige Biotech (Guangzhou, China), are summarized as follows: β-actin: forward (5'-AATCT GGCACCACACCTTCTAC-3') and reverse (5'-ATAGCACAGCCTGGATAGCAAC-3'); IFI27: forward (5'-GCCTCTGCTCTCACCTCATC-3') and reverse (5'-TGGCCACAACTCCTCCAAT C-3'); FOXP3: forward (5'-CCTGGTTGTGAGAAGGTCTTCG-3') and reverse (5'-TGCTCCA GAGACTGCACCACTT-3').

Western blotting analysis

Cells were lysed using RIPA lysis buffer (Tianya Biotechnology, Guangzhou, China) for protein extraction. After determining the concentration with the NCM BCA Protein Assay Kit (NCM Biotech, Suzhou, China), an equal amount of protein was loaded into the electrophoresis chamber filled with SDS-polyacrylamide gel solution. Following electrophoresis, the proteins were transferred to a nitrocellulose (NC) membrane (Merck, Germany). Subsequently, the NC membrane was blocked with a 5% skimmed milk solution for 1 hour and incubated overnight at 4°C with a specific primary antibody dilution. On the second day, the NC membrane was exposed to the secondary antibody dilution at room temperature for 1 h. Subsequently, the NC membrane was washed three times with PBS. Protein signal intensity was detected using the ECL kit (FDbio Science, Hangzhou, China). Acquisition of the signals applied to the strips was performed by a multifunctional gel imaging system (UVITEC,England). For tissue-derived protein blotting experiments, protein lysate from tissues treated with liquid nitrogen freezing and grinding was added, following the same procedure as for cells. The antibodies used in the experiment were as

follows: anti-IFI27 antibody (Cat#NB300-709, Novus Biologicals, USA); anti-FOXP3 antibody (Cat#AF6544, Affinity Biosciences, Australia); anti-E-cadherin antibody (Cat#20874-1-AP, Proteintech, Wuhan, China); anti-N-cadherin antibody (Cat#22018-1-AP, Proteintech, Wuhan, China); anti-Vimentin antibody (Cat#10366-1-AP, Proteintech, Wuhan, China); anti-GAPDH antibody (Cat#10494-1-AP, Proteintech, Wuhan, China).

Immunohistochemistry

Tissue samples were fixed in 4% paraformaldehyde, embedded in paraffin, and sliced continuously into 5μm-thick sections. Subsequently, the paraffin sections underwent dewaxing, hydration, and were transferred to citrate buffer (pH 6.0) for antigen recovery using an autoclave repair method. After a 15-minute incubation with 3% H_2O_2 at room temperature in the dark to inactivate endogenous peroxidase activity, the tissue sections were then blocked in 5% BSA solution for 1 hour. The tissue sections then specifically reacted with primary antibodies. The following day, sequential steps included incubation with the secondary antibody, DAB reagent for color reaction, 3-minute counterstaining with hematoxylin, brief immersion in a 1% hydrochloric acid-alcohol solution for 1 second, 5-minute counterstaining with PBS, dehydration, and mounting with neutral resin. Immunohistochemical staining effectiveness was assessed by calculating the product of the percentage of positive cells and immunostaining intensity. The percentage of positive cells was counted using Image J software, and staining scores were evaluated using a rapid scoring system based on intensity (0, no staining; 1, weak staining; 2, moderate staining; 3, strong staining). Antibodies used: IFI27 (Cat#NB300-709, Novus Biologicals, USA, dilution 1:50) and FOXP3 (Cat#A

F6544, Affinity Biosciences, Australia, dilution 1:100).

Immunofluorescence analysis

Cells were fixed on the cell crawls with 4% paraformaldehyde for 10 minutes at room temperature following the experimental protocol. Subsequently, the cells were washed three times with PBS and permeabilized with 0.3% Triton X-100 at 4°C for 2 minutes. After the cells were blocked in 5% BSA solution for 0.5 h, cells were incubated with the primary antibody at 4°C overnight, followed by secondary antibody (Cat#K1212, APExBIO, USA) incubation. Cell nuclei were stained with DAPI, and representative fluorescence images were captured using an AX confocal microscopy (ECLIPSE Ti2-E, Nikon, Japan). Statistical analysis of experimental data was conducted using Image J software (NIH, USA).

Colony formation assay

1×10^3 cells were inoculated into 6-well plates and cultured for one week. The culture medium was then removed, washed three times with PBS buffer, and fixed in 4% paraformaldehyde solution for 15 minutes. After removing the fixative, the cells were washed three additional times with PBS buffer and the cell colonies were captured with a high-resolution camera. The number of cell colonies was statistically analyzed using Image J software (NIH, USA).

Wound-healing assay

Cells were seeded in a 6-well plate, and when the cells were completely spread over the bott om of the plate, the central area of the cell monolayer was carefully scraped with a 10 μL pi pette tip. The cells were then washed three times with PBS buffer to remove floating cells. R epresentative images were taken at different time points (0 h, 48 h) to observe migration and wound closure. Experimental results were analyzed using Image J software (NIH, USA).

Transwell assay

Cell migration ability was assessed using Transwell chambers (8μm; Corning, USA). Cells, in equal numbers, were suspended in serum-free culture medium and transferred to the upper c hamber, while the lower chamber was filled with complete culture medium containing 10% f etal bovine serum. Following a 12-hour incubation period, cells were fixed and stained, and s tatistical images were captured using an inverted light microscope (Leica, Germany). Statistic al analysis of the experimental results was performed using Image J software (NIH, USA).

Subcutaneous xenograft tumor model

The Animal Care and Use Committee of Zhujiang Hospital approved the protocols and procedures for the animal study. BALB/c mice, aged 4-6 weeks, were obtained from Bestest Bio-Tech (Guangdong, China). The BALB/c nude mice were randomly assigned to two groups, each comprising 5 mice. Subsequently, 1×10^6 T24 cells stably overexpressing IFI27 were injected into the axilla of the nude mice to establish a subcutaneous tumor model. Starting on the 7th day post-

injection, assessments of subcutaneous tumor dimensions were conducted every 3 days using the conventional formula for computing tumor volume: volume = (width^2 × length) × 0.5. Tumor progression was monitored using an in vivo imaging system (IVIS Spectrum, PerkinElmer, USA). At the conclusion of the experiment, the nude mice were euthanized, and tumor tissues were collected and weighed.

Footpad-popliteal lymph node metastasis model

5×10^5 T24 cells, stably overexpressing IFI27, were injected into the footpad of nude mice to establish a footpad-popliteal lymph node metastasis model. The nude mice were divided into two groups, each consisting of 4 mice. To monitor popliteal lymph node metastasis in nude mice, an in vivo imaging system (IVIS Spectrum, PerkinElmer, USA) was employed. After t he mice were sacrificed, popliteal lymph nodes were collected for volume and weight measur ements.

Bioinformatics analysis

In bladder tumors, the correlation between IFI27 and CD4$^+$ T cell infiltration was analyze d using data from the TCGA database (https://www.cancer.gov/ccg/research/genome-seque ncing/tcga). Bladder cancer-related transcriptome data from the TCGA (https://www.cance r.gov/ccg/research/genome-sequencing/tcga) were analyzed through bioinformatics using R software. The ggpubr package was utilized to generate all box plots. Tumor immune cel l infiltration analysis was performed using the GSVA package and correlation analysis lol lipop plot was conducted by ggplot2 package. Pan-cancer differential analysis data for F

OXP3 were extracted from the TIMER 2.0 database (http://timer.comp-genomics.org/timer/), and PDCD1 and FOXP3 gene correlation analysis results were obtained from the TISIDB database (http://cis.hku.hk/TISIDB/index.php).

Flow cytometry

C57BL/6 mice were divided into five groups based on the treatment plan, with three mice in each group. To establish the subcutaneous tumor model, 2×10^6 MB49 Vector/IFI27 cells were subcutaneously inoculated into each group of mice. Drug treatment commenced on the 8th day post-injection, with mice in the first and second groups receiving PBS. The remaining groups received PD-1Ab (Catalog No. BE0146, BioXcell, USA, 100 μg, i.v.), gemcitabine (Catalog No. A8437-100, APExBIO, USA, 30 mg/kg; i.v.), or a combination of both drugs. The medication was administered for a total of 6 sessions, and on the 32nd day, spleens, tumors, and peripheral blood were collected from the mice for the preparation of single-cell suspensions. In brief, spleen tissues were minced, filtered through a 70 μm cell filter, and processed into cell suspensions after erythrocyte lysis. For tumor tissues, a mixture of DNase I and collagenase IV digestion solution was added to the ground tumor tissues, followed by centrifugation, filtration, and collection of filtered cell samples. Peripheral blood, collected from the retro-orbital venous plexus, was anticoagulated and subjected to red blood cell lysis for subsequent experiments. The prepared single-cell suspensions, following the flow cytometry antibody staining protocol, were utilized for flow cytometry analysis. The following antibodies were used in the flow cytometry analysis: PerCP-Cy™5.5 Rat Anti-Mouse FOXP3 (Catalog No. 563902, B

D Biosciences, USA); BB515 Rat Anti-Mouse CD25 (Catalog No. 564458, BD Biosciences, USA); FITC Rat Anti-Mouse CD4 (Catalog No. 553729, BD Biosciences, USA); PE-Cy™5 Rat Anti-Mouse CD8a (Catalog No. 553034, BD Biosciences, USA).

Statistical analysis

The statistical analysis of experimental results is conducted using GraphPad Prism 10 software. To assess statistical significance, the unpaired Student t-test is employed for two groups, one-way ANOVA for more than two groups, and the Chi-square test. A p-value <0.05 is deemed statistically significant, and the statistical data are presented as mean ± SD (standard deviation). The significance levels are denoted as follows: * $p < 0.05$, **$p < 0.01$, ***$p < 0.001$, ****$p < 0.0001$.

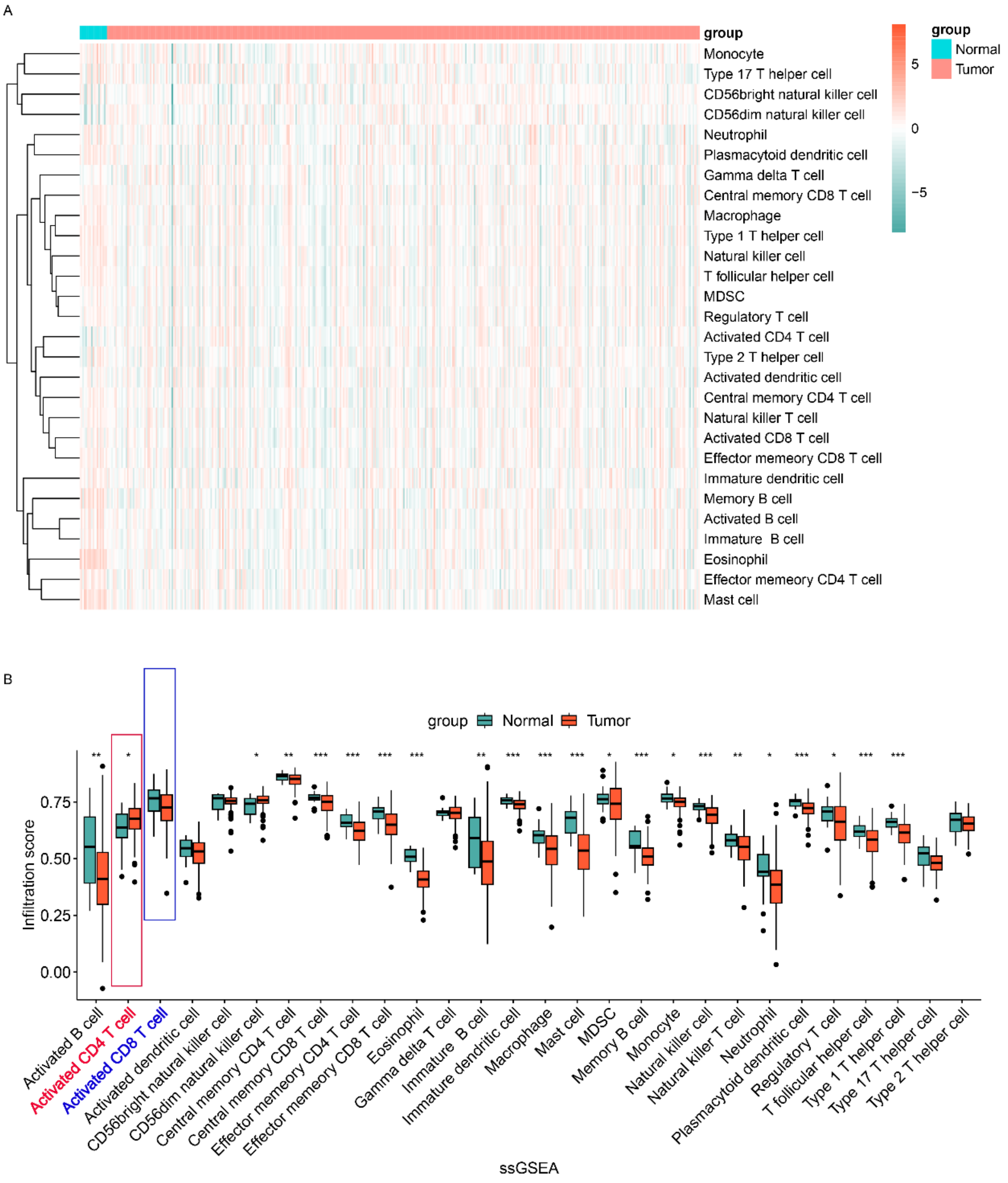

Fig. S1 A Heatmap of the composition of immune cell infiltration in normal bladder tissue samples and BCa tissue samples. **B** Immune cell infiltration scores in normal bladder tissue samples and BCa tissue samples using ssGSEA analysis.

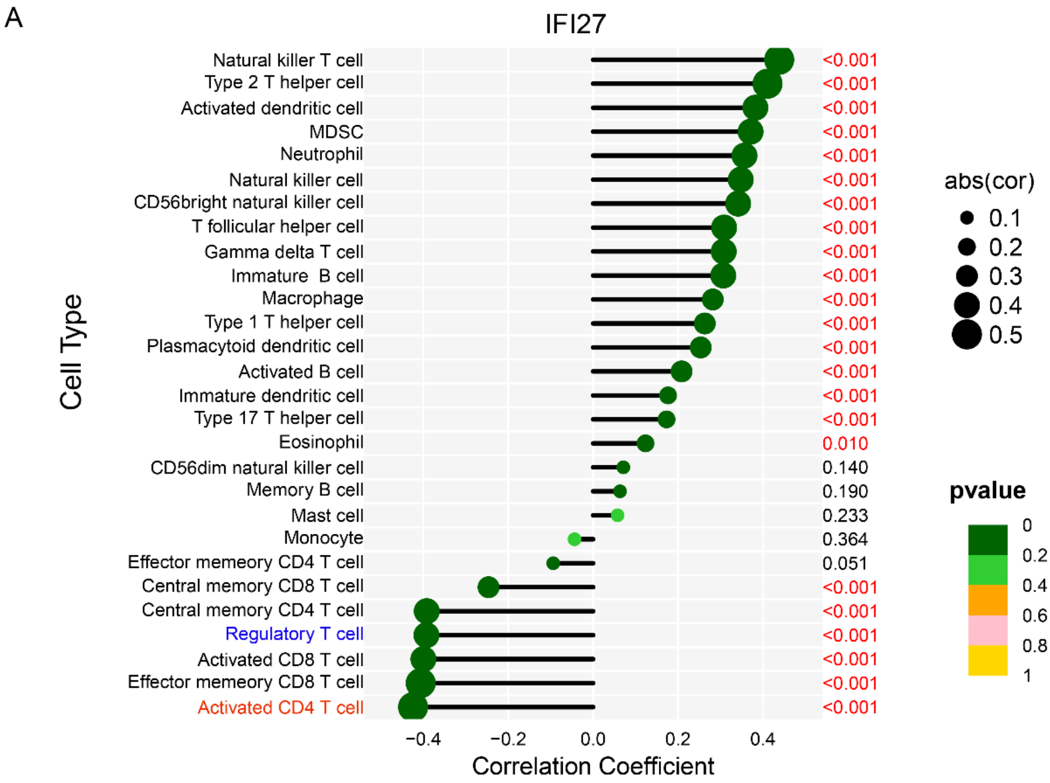

Fig. S2 Lollipop plot showed the correlation analysis between the IFI27 and tumor immune cell populations.

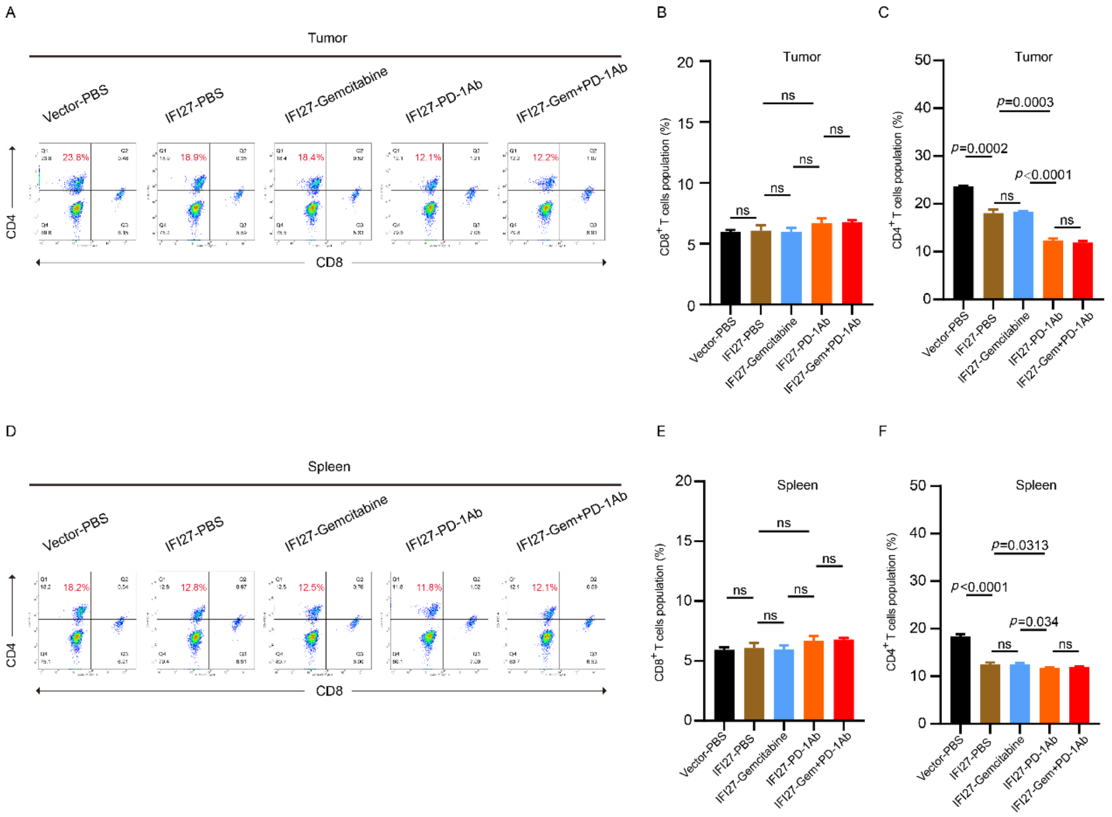

Fig. S3 A-F Flow cytometry representative graphs demonstrated the proportion of $CD8^+$ T cells, $CD4^+$ T cells in mouse tumor (**A**) and spleen (**D**). Statistical analysis showed the differences in $CD8^+$ T cells, $CD4^+$ T cells enrichment in tumor (**B, C**) and spleen (**E, F**) of mice in each group.

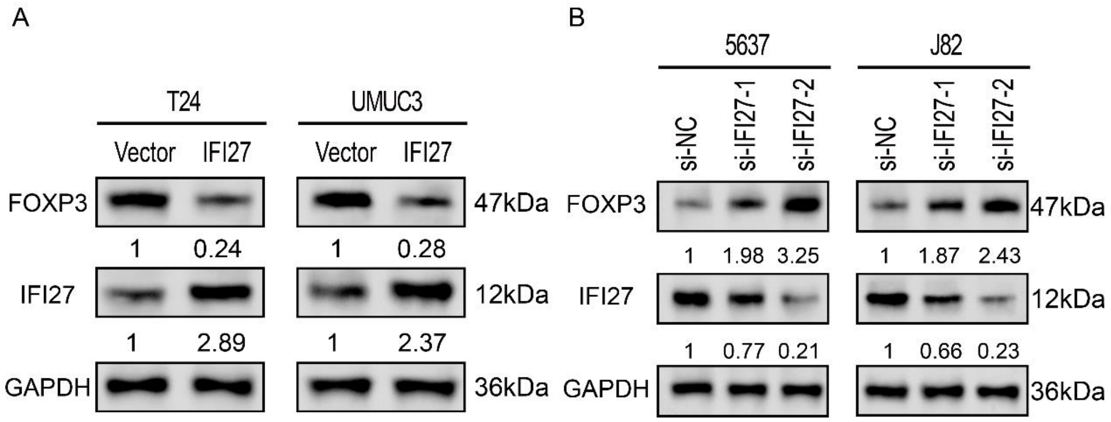

Fig. S4 A Western blotting analysis showed that after overexpression of IFI27, the expression level of FOXP3 was downregulated in T24 and UM-UC-3 cells. **B** Western blotting analysis revealed that after knocking down IFI27, the expression level of FOXP3 was upregulated in 5637 and J82 cells.
